# Oleogels as a Promising Alternative to Animal Fat in Saturated Fat-Reduced Meat Products: A Review

**DOI:** 10.3390/gels10020092

**Published:** 2024-01-25

**Authors:** Md. Jannatul Ferdaus, Bishal Barman, Niaz Mahmud, Roberta Claro da Silva

**Affiliations:** Family and Consumer Sciences Department, College of Agriculture and Environmental Sciences (CAES), North Carolina A&T State University, Greensboro, NC 27411, USA; mferdaus@aggies.ncat.edu (M.J.F.);

**Keywords:** oleogels, meat products, substitution, animal fat, saturated fat

## Abstract

The surge in chronic diseases is closely linked to heightened levels of saturated and trans fatty acids in processed foods, particularly meat products. Addressing this concern, various strategies have been employed to alleviate the impact of these detrimental fats. Among these, oleogels have emerged as a novel and promising approach in the food industry. As restructured fat systems, oleogels offer a unique opportunity to enhance the nutritional profile of meat products while providing distinct health and environmental advantages. This comprehensive review explores the transformative role of oleogels as innovative substitutes for traditional animal fats in a variety of meat products. Utilizing materials such as hydroxypropyl methylcellulose (HPMC), sterols, beeswax, γ-oryzanol, β-sitosterol, and others, oleogels have been investigated in diverse studies. The examination encompasses their impact on the textural, nutritional, and oxidative dimensions of meat patties, pork patties, pork liver pâtés, beef heart patties, and meat batters. An in-depth exploration is undertaken into the influence of various elements, including the type of oil, gelling agents, and processing methods, on the stability and physicochemical attributes of oleogels. Additionally, the paper scrutinizes the potential effects of oleogels on sensory attributes, texture, and the shelf life of meat products. In conclusion, this collective body of research emphasizes the versatility and efficacy of oleogels as viable replacements for traditional animal fats across a spectrum of meat products. The documented improvements in nutritional quality, oxidative stability, and sensory attributes pave the way for the development of healthier and more sustainable formulations in the meat industry.

## 1. Introduction

Dietary fats constitute an essential macronutrient crucial for good health, serving as the most concentrated energy source compared to carbohydrates or proteins [1]. In processed food products, fats significantly enhance organoleptic features such as texture, mouthfeel, and flavor. This macronutrient is broadly categorized into saturated fatty acids (SFAs) and unsaturated fatty acids (UFAs) [2]. Saturated fats exhibit saturation with hydrogen molecules and lack double or triple bonds in their chemical structure. While they serve particular purposes, research has indicated that an excess of saturated fats can contribute to cardiovascular diseases (CVDs) and is recommended to be restricted to below 10% of daily energy intake [3].

Meat products, favored by consumers for their rich content of protein, fat, minerals, carbon polymers, vitamins, and palatable taste, are not without drawbacks. The notable issue lies in their high-fat content, predominantly saturated fat and trans fatty acids, which contribute to desirable functionality, texture, flavor, and mouthfeel [4]. Recognizing the adverse health effects of saturated fat, particularly trans fatty acids, food producers, including those in the meat processing industry, actively strive to reduce these levels. Traditionally, hydrogenated fats are utilized in meat product industries due to the susceptibility of unsaturated plant oils to rancidity during processing [4]. Hydrogenation involves reinforcing chemical bonds, resulting in oils adopting a solid phase, with potential trans fat formation in partial hydrogenation [5].

**Figure 1:**
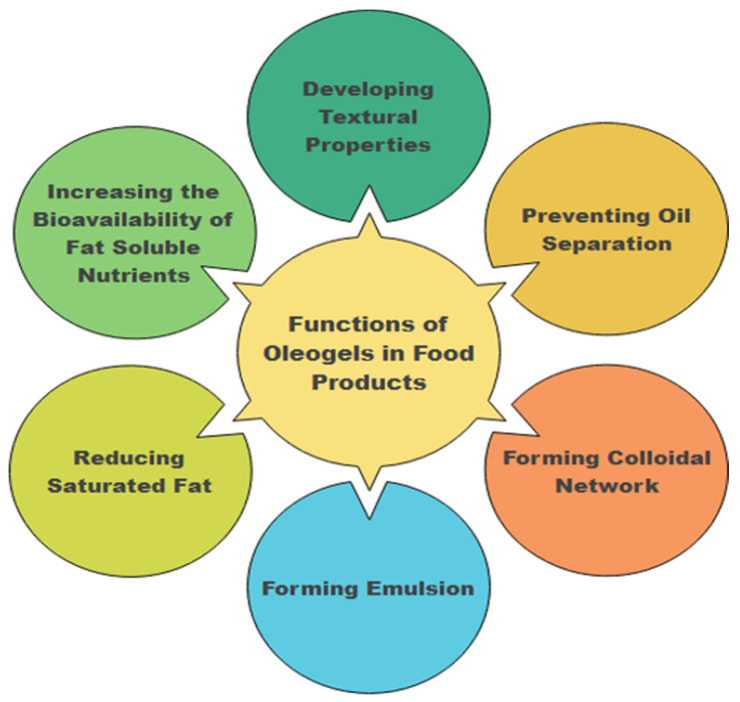
Different functions of oleogels in food products.

Recently, food technologists have explored oleogels as a distinctive alternative to hydrogenated oils or animal fats. Oleogels transform unsaturated-rich plant-based oils into solid-like systems, representing a novel strategy. This transformation is deemed a healthier substitute for saturated and trans fats while meeting consumer expectations for functional and sensory qualities [6]. By incorporating natural waxes, glycerides, alcohols, ethylcellulose, and phytosterols—termed oleogelators—oleogels restructure plant oils, creating a gel network in the oil phase and effectively entrapping liquid oils. The resulting state is semi-solid, firm, spreadable, and viscoelastic, commonly referred to as oleogel [2]. In addition, besides lowering the saturated fatty acids percentage in foods, oleogels also have other functions in foods but not limited to developing textural properties, increasing bioavailability of nutrients, preventing oil separation, forming colloidal network, and forming emulsion (Figure 1).

This review primarily focuses on the developmental process of fat substitution in meat products. Structured vegetable oils (oleogels) exhibit solid-like structural characteristics similar to animal fats. Consequently, the following sections detail the structures, properties, preparation methods, and research progress of oleogels as substitutes for animal fat in meat products.

## 2. What Is Oleogel?

Oleogels, also known as organogels, provide a method for transforming organic oil into a solid or semi-solid substance by entrapping them into a thermo-reversible gel structure (oleogelators). By self-assembling or crystallizing to form a wide range of structures, oleogelators may capture liquid oil and keep it from flowing by creating a three-dimensional network structure and gelatinizing the entire system. Oleogels are created using a variety of oleogelators, each of which results in a unique phase transition mechanism, taking place on the nano- and micro-scales and inducing a new set of macroscopic properties [7]. Many gels only need a minimal quantity of oleogelators and are often characterized as bulk-like fat products since they include a lot of liquid oil, sometimes even >97% [2]. Oleogels’ ability to substitute solid fat in food items and enhance the nutritional value of food products is the motivating factor behind the study and development of these food ingredients. Oleogel can benefit things from low-saturated fatty acids while maintaining their solid features. To substitute solid fats or perform other desired activities, such as controlled release and high bioavailability, a good oleogel should have changeable physical and chemical characteristics for edible purposes [8]. It is vital to choose an appropriate oleogelator to produce qualified food-grade oleogel. A good oleogelator for food applications should have natural sources. Different edible ingredients, including glycerol, fatty acids, organic waxes, and monostearate, have recently been investigated as oleogelators. Research has revealed that a mono-component oleogel cannot realistically imitate the characteristics of conventional solid fats; however, a combination of oleogelators can produce a more desired oleogel with a stronger network [2,9]. Barroso et al. (2020) showed that at 5 and 25 °C, multicomponent gels composed of sunflower wax, glycerol monostearate, and berry wax demonstrated solid-like properties. Notably, the mono-component oleogel consisting of sunflower wax displayed more liquid-like characteristics at both temperatures [9].

Research indicates that a mono-component oleogel, while exhibiting some desirable properties, faces challenges in replicating the multifaceted characteristics of conventional solid fats. Hence, the primary hurdle in developing oleogels for food applications is creating a specific crystal network to meet the required solid fat properties, achieving the requisite structural complexity and stability that saturated fats inherently possess [2].

The oleogelators play a crucial role in controlling the network structure and resultant properties of oleogels. Mono-component gels often lack the intricate network and crystalline arrangements found in saturated fats, leading to suboptimal textural attributes and performance in certain applications. The adverse results observed may include inferior mouthfeel, compromised stability under varying conditions, and challenges in mimicking the specific melting profiles characteristic of saturated fats. Thus, recent research has shifted towards utilizing combinations of oleogelators [2,9].

Recent studies have underscored the significance of employing a combination of oleogelators to address these challenges [3,5,9]. The synergistic interaction between different edible ingredients, each contributing unique structural elements and functionalities, has been found to enhance the overall performance of oleogels. This approach, involving a blend of oleogelators, has shown promise in achieving a more desirable and robust oleogel structure that closely mimics the characteristics of saturated fats. The combination of oleogelators has the potential to strengthen the interfacial bonds, consequently bolstering the oleogels’ resilience to drying, enhancing gel strength, elevating oil binding capacity, and fortifying thermal stability [5].

Oleogels (cellulose derivatives) are thermo-reversible because they can go through the sol–gel transition several times with only the warming and re-cooling of the solution. Different external conditions, including pH, light, and solvent changes, are needed for other oleogels that usually are not employed in food systems to stimulate self-assembly. In apolar liquid, very efficient oleogelators may produce gels at concentrations as low as 0.5 wt%. The capacity to form oleogels at low concentrations is attractive for the food industry [10]. Although oleogels are still being developed for food applications, the cosmetics and pharmaceutical sectors have extensively researched them.

## 3. Strategies in Oleogel Preparation

### 3.1. Crystal Particle Systems

**Figure 2:**
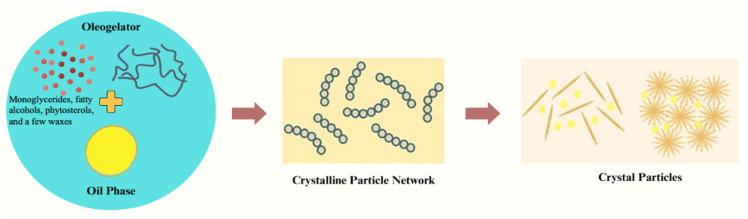
Overview of crystalline particle oleogelation system.

The packing and organization of the structuring agent particles in the oleogel network are referred to as the crystal particle system of oleogelation. The physical characteristics of the oleogel are influenced by the arrangement of these particles, which can be either crystalline or amorphous (Figure 2). Oleogels can form various crystal particle systems, including lamellar, cubic, hexagonal, and others [11]. Several variables, including the kind of oil and the oleogel-making conditions, influence the type of system that develops. In this system, the structuring agents can crystallize, including monoglycerides (MG), fatty alcohols, phytosterols (PS), and a few waxes [12]. Nano molecules of structuring agents produce a three-dimensional matrix of crystals. The main factor in forming a network in oleogels is the crystallization behavior of the oleogelator. The crystals grow into clusters in cool temperature; they form a tight crystalline network, keeping the liquid oil inside the network structure. Like typical solid fats, these oleogelators act by a process that prevents plant oil from flowing through the crystal network, giving the final product gel-like properties [11]. However, these crystals’ patterns, morphologies, and characteristics distinguish them from those of conventional solid fats. Oleogelators like candelilla wax (CLW) and MG often produce one- or two-dimensional crystal structures. These oleogelators may create a dense crystal network to gel the oil phase at lower concentrations. Wax lipids of saturated wax ester acid with carbon chain lengths of 10–31 can create oleogel structures. The lowest concentration required for gel formation in liquid oils and fats lowers as the chain length grows [13]. In contrast to saturated glycerides, wax esters found in edible oils exhibit the formation of flakes or needles of crystals, even at low concentrations. The waxes have the capability to assemble into a crystalline structure, thereby restraining the liquid oil and giving rise to the formation of an oleogel. The crystal network structure of the oleogel becomes more intricate and divides into numerous crystal aggregates with the incorporation of waxes. Wax, a fatty substance characterized by lengthy hydrocarbon chains, is produced by animals and plants. Some widely utilized bio-based waxes, including carnauba wax (CRW), rice bran wax (RBW), beeswax (BW), and candelilla wax (CLW), are recognized for their accessibility and applicability in various food products. These waxes are easy to acquire and can shape liquid oil into an edible oleogel at low concentrations [11]. According to research, canola oil might be structured by RBW, CLW, and CRW at concentrations of 1%, 2%, and 4%, respectively [14]. Because of its smaller crystal size and more uniform crystal spatial distribution, CLW oleogel was shown to have a higher oil-binding capacity than RBW and CRW oleogel. In a different study, RBW required more than 5% w/w to structure rice bran oil, while BW and CLW showed low critical gelling concentrations (less than 2%) [15]. Additionally, the physicochemical characteristics of oleogel made from various waxes fluctuate greatly. For instance, Lim et al. (2017) assessed bio-based waxes’ texture, thermal rheology, and oxidation properties in canola oil oleogels [16]. Following CRW and BW samples in order of increasing hardness, BW oleogels had the best adhesive and cohesive properties. The lowest peroxide values were also seen in the CLW oleogels, indicating that the firmer texture was associated with a stronger antioxidant capacity.

The utilization of diverse oleogelators in the preparation of oleogels has gained increased attention in recent times. For instance, although gels created with a single oleogelator are unstable and susceptible to polymorphic change, MG and PS may each make oleogels with edible oil. The mixed component oleogels, on the other hand, improved stability and added excellence, further simulating and replacing solid fats [17]. In recent research, the percentage of these oleogelators was changed to create and define smooth MG and PS oleogels. According to the findings, MG and PS have resulted in better gel networks, and oleogels with an 8:2 ratio showed the best qualities [12]. According to another research, using MG and PS combined to make oleogel resulted in a high amount of polyunsaturated fatty acid (PUFA), successfully replicating the functioning of pork back fat [18]. In another study [19], using adipic acid (AA), it was possible to increase the network strength by inducing crystallinity.

The micrograph of the oleogel containing 6% ethylcellulose portrayed a compact and irregular network, indicative of efficient oil entrapment. This observation resonates with a previous study [8] that reported extensive polymer networks in EC-based oleogels. Contrastingly, the microstructure of the oleogel exclusively formed with adipic acid exhibited a distinctive needle-like crystal structure, reminiscent of networks documented in prior research on fatty acid-based oleogels [20]. Introducing adipic acid into both EC and the EC/AA combination oleogels significantly altered microstructure. The spatial hindrance created by AA crystals reduced polymer network extension, a phenomenon influenced by the ratio of oleogelators. No significant changes were observed in the microstructures up to a 2% AA concentration, while higher concentrations increased the number of crystal structures in the oleogel network. This observation aligns with the outcomes of XRD analyses, highlighting the influence of free AA at higher concentrations. The phenomenon can be attributed to a reduction in aggregation and an improvement in the distribution of crystals at lower EC concentrations [19]. Consequently, this oleogel has demonstrated its suitability for application in bakery and meat products, emerging as a promising substitute for hydrogenated shortening and animal fat.

Several experiments have examined multicomponent oleogel systems to see how well the oleogelators work together. Yang et al. (2020) created and described the mixed component gel to study the beneficial connection involving monopalmitate (MP) and CRW. The multicomponent oleogel differed from the MP (or CRW) oleogel in terms of both the apparent crystallization constant and the Avrami exponent. As a result, the characteristics and crystallization of oleogels were affected by the synergistic interaction of MP and CRW. Further, ethylcellulose (EC) changed the gel’s crystallization characteristics when added to the stearyl alcohol and stearic acid, creating a synergistic system [20].

### 3.2. Self-Assembly Systems

**Figure 3:**
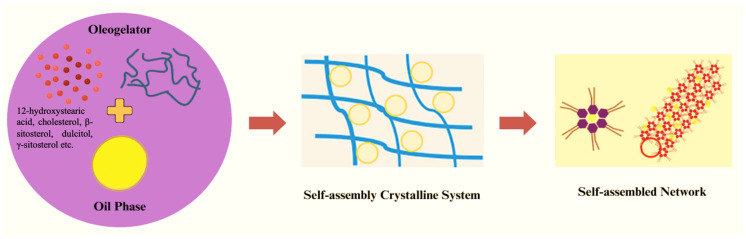
Overview of self-assembly oleogelation system.

The initial self-assembly of oleogelators occurs within the oil phase, initiating a supersaturation phenomenon when the solvent’s melting point is lower than that of the oleogelator. This results in a nucleation process where oleogelator molecules form basic structural units (Figure 3). Subsequent one-dimensional growth, helix formation, and torsion contribute to developing a fibrous network structure, restricting the movement of liquid oil and facilitating the gelation of the entire system [21].

All different types of oleogelators have self-assembly properties, and at some point, they show it in oleogelation. Structuring agents like 12-hydroxystearic acid create spiral or twisted crystalline filaments to impede oil movement. Molecular interactions, such as hydrogen bonding, van der Waals forces, dipole, and hydrophobic forces, mimic the self-assembly and crystallization of triacylglycerols [22]. A previous study by Sawalha et al. (2015) examined the correlation between gel-forming characteristics and the molecular structure of the oil-forming agent by employing dihydrocholesterol, cholesterol, β-sitosterol, and dulcitol. These substances were utilized to self-assembly into robust and clear gels with γ-sitosterol. The study reported that the ability to form gels is predominantly contingent on the hydrogen bonding interactions between the hydroxyl group of sitosterol and the carbonyl group of oryzanol. Additionally, this gelation capacity is influenced by the degree of desaturation in the cholesterol ring structure [23].

Recent studies explored structuring edible oils using a combination of γ-oryzanol and β-sitosterol. Mechanical treatment, such as shear, enhanced oleogelation rather than immediate gelation after cooling—β-sitosterol and γ-oryzanol act as capillaries between self-assembled co-crystals, trapping oil within the oil structure. The structuring units of both γ-oryzanol and β-sitosterol docked on top of each other at the molecular level, generating thin fibrillar building blocks [24]. Calligaris et al. (2014) investigated oil sources’ gelation duration, thermal properties, and network structure attributes with increasing viscosity and polarity using combinations of γ-oryzanol and β-sitosterol [25]. In a separate study, small-angle X-ray scattering supported the hollow fibrillar form and self-assembly process of the mixed oleogel of β-sitosterol and γ-oryzanol, demonstrating its capacity to provide structure to oil [26].

### 3.3. Polymeric Network

**Figure 4:**
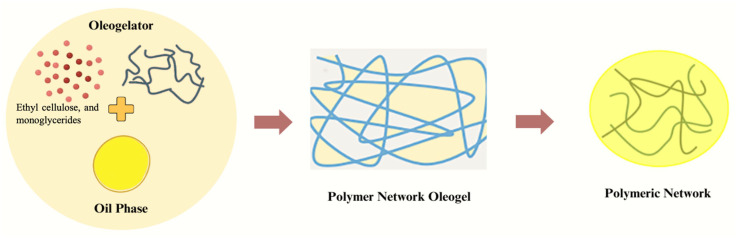
Overview of polymer network oleogelation system.

The formation of polymeric oleogels through cross-linking or self-assembly by chemical bonding is a crucial process involving structural molecules (Figure 4). Ethyl cellulose (EC) is the singular polymeric gelling agent capable of direct dispersion in oil. Its versatility allows modification for specific needs, and its semi-crystalline and hydrophobic properties make it particularly suitable for oil-gelling agents. Due to its semi-crystalline nature, the sol–gel transition of EC in the presence of liquid oil involves the phase transition temperature, causing the EC polymer to melt and disperse in the oil. During cooling, the cross-linking of ECs in the hydrophobic phase creates a homogeneous and stable three-dimensional network structure, binding the hydrophobic phase oil through physical factors such as hydrogen bonds and van der Waals forces [27].

In a study by Zhang et al. (2019), the high-temperature cross-linking of EC molecules in cinnamon oil oleogels revealed a denser system with increased EC viscosity, enhancing oil binding and stability [28]. Combining EC with oleogels of monoglycerides (MG) and candlestick wax (CW) improved rheological characteristics by increasing EC solubility and establishing hydrogen bonds between the hydroxyl groups of EC and MG.

Specifically, EC, among all polymer oleogelators, demonstrates the unique ability to structure oil directly. Gelation converts some crystalline regions into amorphous parts, exposing ethoxy groups and forming a coral-like polymer network during a phase transition. This network structure, reliant on intermolecular hydrogen bonding, weakens in real-life conditions [29]. Recent investigations on EC oleogels have explored the impact of solvent polarity, heat treatment, and surfactant addition on mechanical properties, highlighting the role of hydrogen-bonding networks. As Davidovich-Pinhas et al. (2016) studied, thermal application indicated a decrease in storage modulus with an increasing temperature, underscoring the importance of hydrogen bonding in EC gel network development [30].

Solvent polarity emerges as a significant factor influencing the mechanical characteristics of EC oleogels, as polar functional groups interact with polymer chains, creating hydrogen bonds in the lipid phase. Surfactant addition, as observed by Davidovich-Pinhas et al. (2015), significantly impacts the gel’s structural behavior, with surfactant–polymer interactions further stiffening the network [31]. The intricate interplay between the oleogel’s polymer and solvent significantly influences the structural behavior of the gel under different conditions.

### 3.4. Indirect Templated System

**Figure 5:**
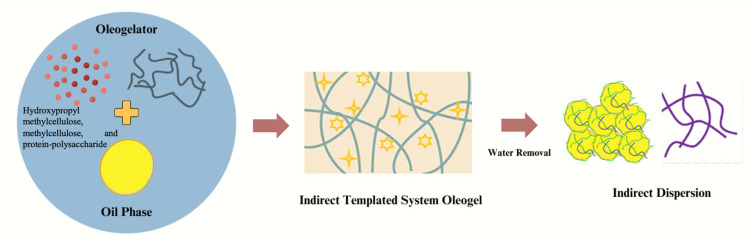
Overview of indirect templated oleogelation system.

Direct oleogel reactions have drawbacks since the high-temperature preparation causes oil to oxidize and lose bulk. The mechanism of indirect templated system has been illustrated in Figure 5. To enhance the production of oleogel, for instance, using EC in the direct gelation process requires a high heating temperature of over 100 °C, which might cause the oil to degrade [12]. As a result, several researchers have investigated making oleogels using indirect template techniques, primarily emulsion, foam, or solvent exchange techniques [8,17]. The emulsion is often employed as a template for the manufacture of oleogel in indirect gel processes. Following specific treatments, the specialized procedure creates an emulsion with high oil content, and the interaction of the polymer of emulsifiers in the droplet creates a network structure in the emulsion system. The liquid phase stays in the gel network, while the continuous phase comprises solid fat crystals. After that, the water is entirely removed from the continuous phase, leaving behind a polymer network in which the oil droplets are securely packed [8]. The development of oleogel may then be induced by shearing the polymer network. In a recent study by Espert et al. (2020), the authors looked at the viability of using an emulsion template approach to use hydroxypropyl methylcellulose (HPMC) and methylcellulose (MC) as gelators to create sunflower oil oleogels [32]. This study shows that MC and HPMC may use the indirect template approach to shape oil. Further, results showed that the initial oil and cellulose ether concentrations were essential in oleogel physical behaviors [32]. Another comparative research that employed various concentrations of HPMC to create oleogels using the emulsion template approach similarly supported the earlier finding. With an increase in HPMC content, the gel produced showed a stronger mechanical structure and higher ability to bind oil, as high concentrations of HPMC created multilayers that offered better protection for oil droplets [9]. By absorbing liquid oil into a porous template made of a dry polymeric network, oleogels may also be produced [32]. Aerogel has recently been suggested as an oleogel template. Plazzotta et al. (2020) reported successful oleogel production using an aerogel template [33]. In this instance, the carrageenan-containing hydrogel was first transformed into an alcohol gel, which was then dried using supercritical carbon dioxide to produce aerogels. Finally, oleogels were created using aerogels as a template. The results showed that aerogels could make oleogels, in which oil content and retention depended on the structural organization of the aerogel, as indicated by the number, size, and length of the pores. In related work, Manzocco et al. (2021) created oleogel by mixing oil with aerogel, producing either freeze-drying or supercritical carbon dioxide drying [34]. Supercritical carbon dioxide-dried aerogel produced a stronger, more plastic oleogel than the latter aerogel did, and the resulting oleogel had many rheological characteristics with conventional fats. In the research indicated above, porous templates were created using the available oleogelators to encourage the creation of oleogels. A recent study made it possible to use protein-polysaccharide conjugates to create an aerogel template for oleogel. The alginate/soy protein conjugates were made via the Maillard process, freeze-dried to create the aerogel templates, and then submerged in maize oil for 6 h to develop the oleogels.

However, the drawback of the indirect technique of gel production is the need to dry the oleogel sample to stop the aqueous phase and introduce other components, such as proteins, during the preparation process. However, dehydration may result in the oil phase aggregating and the oxidation of unsaturated oils owing to interface destabilization, which is detrimental to the creation and stability of oleogel systems.

## 4. Oleogel Applications in Meat Product

Modifying processed meat’s quantities and lipid profiles, pivotal sources of dietary fat, can enhance the nutritional quality of the Western diet [35]. Reformulation, a critical approach involving removing, reducing, adding, or replacing various components, stands out as a key method for crafting healthier meat products. Through reformulation, key objectives in fat content improvement include reducing total fat and energy content, lowering cholesterol, and modifying fatty acid profiles [7,36]. While technological methods often replace animal fat to optimize fatty acid profiles, challenges arise in maintaining essential product qualities (mouthfeel, juiciness, texture, bite, heat transmission) associated with the solid animal fat in meat products. This poses a significant technological challenge when attempting to reduce or substitute animal fat with liquid oils [37]. Despite numerous experiments and efforts to enhance the fat content of meat products and find alternatives to animal fat, the use of technical methods to replace non-meat fat with animal fat has been extensively explored. Some previous studies have explored diverse avenues, including using various vegetable oils, marine oils (from fish and algae), or combinations to partially substitute animal fat in fresh, cooked, and fermented meat products [38,39]. Notably, there is limited research on using oleogels for developing healthier, lipid-rich meat products. Some recent studies have begun investigating the potential applications of oleogel systems in various meat products, such as meat burgers, batters, patties and frankfurters, fermented sausages, and pâté (refer to Table 1). This emerging research explores oleogels as a novel approach to developing nutritious meat products.

### 4.1. Burgers

Since it enhances the meat’s flavor, texture, and juiciness, animal fat is crucial in burgers. The fat gives the burger moisture, which keeps it moist and tasty while cooking. The fat also helps keep the beef together, preventing the patty from disintegrating [40]. The type of animal fat utilized can also impact the burger’s flavor. For instance, beef fat has a distinctive flavor that might improve the taste of the meat. It is crucial to remember that consuming too much animal fat can have adverse health effects [1], so eating burgers in moderation is preferable as part of a healthy diet.

Gómez-Estaca et al. (2019) aimed to evaluate the sustainability of oleogels derived from ethyl cellulose (EC) and beeswax (BW) obtained through a blend of olive, linseed, and fish oils [39]. These oleogels were explored as alternatives to conventional fats in low-fat, polyunsaturated fatty acid (PUFA)-enriched pork burgers. The oleogelation process involved EC contributing to a “polymeric network” and beeswax functioning within a “crystal particle system,” resulting in a semi-solid phase. Compared to the control group, where the developed oleogels entirely replaced traditional pork back fat, the low-fat pork burgers exhibited a notably softer texture without significant changes in optical properties. Moreover, the reformulated burgers demonstrated substantial improvements in the fatty acid profile, presenting a remarkable 3.6-fold increase in the PUFA/SFA ratio and a significant 23-fold reduction in the n-6/n-3 ratio compared to the control group. The oleogels’ stability was maintained for at least one month when stored at 3 ± 1 °C. Sensory acceptability assessments indicated that burgers made with beeswax oleogels consistently received high ratings, while those prepared with EC scored below the neutral rating threshold. In conclusion, the findings suggest that these oleogels have the potential to produce healthier fresh pork burgers with an improved fatty acid profile.

In a study by Moghtadaei et al., 2018, the objective was to develop beef burgers with varying levels of animal fat substitution using oleogel and assess their physicochemical properties [41]. Sesame oil oleogels were synthesized with beeswax (BW) at 5%, 7.5%, and 10% concentrations. Subsequently, 0%, 25%, and 50% of animal fat in beef burgers was replaced with the oleogel. Various characteristics of both control and formulated burger samples were analyzed to assess the impact of oleogel addition. The results revealed that as the level of animal fat replacement increased with the oleogel, the moisture content increased while the fat content decreased. Higher moisture content in food products is associated with lower oil absorption during frying, potentially explaining the reduced fat content in burgers with a 50% oleogel replacement. In raw burgers, the hardness decreased as the level of animal fat was substituted due to the smaller size of fat globules. The addition of BW oleogel significantly reduced the lightness of raw burgers. Cooking loss was most pronounced in burgers produced with animal fat, but cooking loss decreased as the replacement level increased. An increasing oleogel concentration in the burger correlated with a 1.5% reduction in fat absorption. In the oxidative stability test, the sample produced with animal fat exhibited the lowest TBARs value, and as the oleogel concentration increased, TBARs values rose. Sensory evaluation results indicated that panelists could not detect differences in texture and color among the burgers; however, they preferred the flavor of burgers produced with BW oleogel. This comprehensive study provides valuable insights into beef burgers’ physicochemical attributes and sensory aspects, incorporating oleogel as an animal fat substitute.

A previous study by Gómez-Estaca et al. (2020) examined how household activities, including refrigerated storage and cooking, impact the quality attributes of reduced-fat PUFA-enriched pork burgers [42]. Beyond household activities, they explored the influence of an oleogelation system involving an olive–linseed–fish oil blend and the integration of curcumin (CU) as an antioxidant, focusing on health implications. A comparative analysis with a control sample composed of pork back fat revealed that samples formulated with BW, BW-CU, EC, and EC-CU exhibited elevated levels of PUFA and MUFA. Furthermore, these formulated samples demonstrated significantly reduced levels of SFA, resulting in a higher PUFA/SFA ratio and a noteworthy decrease in the n-6/n-3 ratio—notably, the oleogel-incorporated samples delivered approximately twice the calories of the control counterparts. The utilization of oleogel contributed to a softer texture in the burgers compared to the control. Overall, the sensory attributes of burgers incorporating BW oleogel were deemed satisfactory and showed less lipid oxidation on storage.

Further, Moghtadaei et al. (2021) conducted a study investigating the partial substitution of animal fat with ethyl cellulose (EC) oleogel in beef burgers [43]. The oleogels, essential for substitution, were formed by combining sesame oil with EC. Compared to animal fats, EC oleogel exhibited higher concentrations of linoleic and linolenic acids, lower levels of palmitic and stearic acids, and an absence of myristic acid. As the EC oleogel content increased in the burger samples, the moisture content increased while the fat content decreased. EC oleogel seemed to create more fat globules, engaging additional proteins surrounding them. Cooked burgers with oleogel substitution demonstrated a roughly two-fold decrease in saturated fat content compared to control samples without animal fat replacement. However, oxidative stability was reduced in oleogel-incorporated samples. The study revealed that the hardness of raw beef burgers with EC oleogel was lower than those prepared with animal fat. Interestingly, an alternative hard behavior was observed in cooked samples, attributed to hydrogen bond breakage during temperature elevation, enabling additional protein interaction and resulting in a firmer texture. Sensory acceptability tests, encompassing color, taste, and texture, indicated that burgers produced with EC oleogels received higher ratings. The panelists favored beef burgers with a higher oleogel concentration (50%). This research suggests that oleogels hold the potential for substituting animal fat in meat products, enhancing textural qualities while reducing overall fat content.

### 4.2. Sausages and Frankfurters

Sausage, a popular meat product commonly prepared with pork and fat, is frequently consumed as a breakfast item. The composition and texture of sausage products heavily depend on fat as a key ingredient, exerting a profound influence on their sensory attributes. Fat, muscle tissue, and water are pivotal in determining emulsion stability in meat sausages. Consequently, understanding the potential impact of variations in fat quality is imperative for assessing and maintaining the overall quality of sausage products.

Barbut et al. (2016) explored the potential of structuring canola oil to replace animal fat in breakfast sausages while preserving textural and sensory properties [38]. This study aimed to evaluate the impact of substituting pork back fat with linseed oleogel on the key quality parameters of Frankfurters. An oleogel was created by incorporating 8% (*w*/*w*) beeswax into the linseed oil phase as a pork fat substitute in frankfurter sausage. Three sausage batches were produced: a control batch with pork back fat and two with 25% and 50% of pork back fat replaced by oleogel, respectively. Oleogels were prepared using canola oil, EC (8, 10, 12, and 14%), and sorbitan monostearate (SMS) (1.5% and 3%). The sausages underwent testing for texture profile, cooking loss, color assessment, microstructure, and sensory properties. Pork breakfast sausages produced with SMS-free oleogels exhibited lower objective hardness and springiness ratings but comparable cohesiveness to controls containing pork fat and canola oil. However, incorporating SMS into the oleogels resulted in accurate hardness values closely resembling those of the pork fat control treatment. This indicates that utilizing oleogels holds promising potential for improving the texture of sausages in the meat industry. Furthermore, creating tougher oleogels allows the customization of hardness qualities, considering various manufacturing-related parameters. This study underscores the viability of oleogels as a valuable tool for enhancing the textural attributes of sausages.

The investigation by Wolfer et al. in 2018 aimed to assess the influence of substituting pork back fat with soybean oil oleogel, structured with varying levels of rice bran wax, on the sensory characteristics of Frankfurter-type sausages [44]. The introduction of soybean-based oleogel elevated polyunsaturated fatty acids (PUFA) levels, particularly linoleic and α-linolenic acids. Notably, despite soybean oil’s limited n-3 fatty acid content, it improved the n-6/n-3 fatty acid ratio in the oleogel system. Frankfurters with oleogels exhibited increased puncture resistance, which can be attributed to oleogels’ ability to maintain structural integrity during manufacturing. This results in larger fat globules requiring higher energy input for reshaping. Oleogel-formulated samples demonstrated uniform hardness values, except those introduced later displayed elevated hardness. The 10% rice bran wax (RBW) oleogel-integrated sample exhibited extensive lipid oxidation, possibly accelerated during the prolonged heating needed for wax dissolution—a crucial step in 10% RBW. Distinct “Plastic” or “Grassy” sensory attributes were noted in 10% RBW oleogel samples, while higher oleogel content correlated with increased chewiness and hardness. The study highlighted oleogel technology as a viable alternative to animal-derived fats in processed meat products. Oleogel integration, replacing traditional fats, yielded Frankfurters with satisfactory technical attributes and notably enhanced nutritional composition, evidenced by the favorable fatty acid profile in oleogel-infused sausages.

In a study conducted by Franco et al. in 2019, linseed oil was structured with beeswax (BW) to create an oleogel aimed at assessing the impact of substituting pork back fat on the physicochemical, nutritional, and sensory characteristics of Frankfurter sausages [45]. Two replacement levels, 25% and 50%, were examined. Both replacements exhibited a significant reduction in cholesterol levels. Across all batches, MUFA, SFA, and PUFA consistently dominated the fatty acid profile. Particularly noteworthy was the substantial decrease in SFA content, specifically palmitic and stearic acids, indicating an overall improvement in the fatty acid profile. The 25% and 50% replacements demonstrated a PUFA increase of approximately 19% and 51%, respectively. The 50% replacement reduced the n-6/n-3 ratio from 14.92 to 1.61 and increased the PUFA/SFA ratio from 0.47 to 0.78 compared to the control. Moreover, the incremental inclusion of oleogel intensified the yellow coloration of the reformulated sausages. Textural metrics, including hardness, were nearly identical to the control. In sensory evaluations between the 25% and 50% replacements, the control sample was preferred, with panelists attributing this preference to superior visual, olfactory, and gustatory qualities linked to lower lipid oxidation levels than the reformulated counterparts. While the incorporation of linseed oleogels did not yield significant improvements in color and specific sensory attributes, a notable enhancement was observed in the fatty acid composition of the restructured product. Further research is imperative to refine strategies for mitigating lipid oxidation and optimizing sensory attributes in food formulations utilizing linseed oleogels.

In a thorough investigation conducted by Da Silva et al. in 2019, the focus was placed on a comprehensive analysis of Bologna-type sausages, specifically exploring the strategic replacement of pork back fat with high oleic sunflower oil (HOSO) oleogel at varying levels [46]. The preparation of the oleogel itself involved a meticulous blend of pork skin, water, and HOSO in a balanced ratio of 1.5:1.5:1, resulting in an ingeniously crafted oleogel with elevated protein content and a substantial presence of monounsaturated fatty acids (MUFA). The principal outcome of this innovative substitution strategy was a noteworthy reduction in the sausages’ fat content. Increasing levels of HOSO oleogel substitution correlated with a significant decrease in fat levels, with all substituted samples exhibiting an approximate 10% reduction in cholesterol levels—a commendable enhancement. Beyond compositional changes, the substitution of pork back fat induced alterations in textural properties. This transformation was attributed to collagen converting into gelatin, solidifying upon cooling and contributing to a firmer texture in the modified sausages. Crucially, the modified sausages demonstrated lower readings of TBARS, indicating reduced malondialdehyde levels. This was attributed to the decreased lipid content in these samples, coupled with a reduced proportion of polyunsaturated fatty acids (PUFA) in the lipid composition. Notably, at substitution levels of 75% and 100%, there was a discernible reduction in stearic acid concentration. Furthermore, when employing pork back fat substitutions of 50%, 75%, and 100%, a significant decrease of over 30% in saturated fatty acid (SFA) content was observed. Sensory evaluations revealed that substitutions of 25% and 50% of pork back fat with HOSO oleogel did not yield significant differences in sensory characteristics compared to the control group. In conclusion, this study emphasizes that substituting 50% of pork back fat with HOSO oleogel represents a pivotal threshold for enhancing Bologna-type sausage’s nutritional and technological attributes.

In a previous study conducted by Igenbayev et al., 2023, the viability of substituting traditional pork fat in semi-smoked sausages with a sunflower oil oleogel, structured using a blend of beeswax and monoglyceride in a 2:1 ratio, was thoroughly assessed [47]. The formulated oleogel exhibited a total fatty acid content of approximately 62.61% polyunsaturated fatty acids (PUFA), with linoleic acid constituting around 99.3% of the total PUFA content. The replacement of pork fat with oleogel significantly reduced stearic acid in the lipid-formulated semi-smoked sausages, reaching over a two-fold decrease with a 10% and 7% substitution, respectively. Additionally, the incorporation of oleogels led to a substantial 3.5-fold increase in the linoleic acid content of the pork fat-substituted sausages. Notably, the former exhibited superior sensorial attributes when comparing sausages with 7% pork fat substitution using oleogels to their 10% counterparts. The study elucidated the impact of beeswax’s hydrophobic properties on juiciness, monoglycerides’ influence on lipid distribution within the sausage matrix, and the potential instability of the matrix when 10% oleogel was incorporated, affecting overall appeal. These findings underscore that substituting pork fat with oleogel can significantly alter the fatty acid composition of sausages, thereby influencing their nutritional profile. Consequently, the study concludes that the strategic incorporation of oleogel as a partial replacement for pork fat is a scientifically sound approach to enhancing the nutritional quality of sausages, resulting in improved fatty acid profiles and sensory attributes. This approach promotes the overall healthful characteristics of the sausages.

The impact of substituting pork back fat with an oleogel derived from peanut oil (PO) structured by ethyl cellulose (EC) was explored by Shao et al. (2023) in Harbin red sausages [48]. Comparative analyses were conducted on traditional pork back fat and oleogel-substituted samples across various parameters. The modified samples consistently displayed a notable reduction in total lipid content, with the 50% substitution variant registering the lowest total fat level. As the percentage of oleogel substitution increased, there was a consistent decline in saturated fatty acid (SFA) content and a significant rise in unsaturated fatty acid (UFA) content, peaking at approximately 22% in the 50% substitution sample. Moreover, the polyunsaturated fatty acid (PUFA)-to-SFA ratio in oleogel-incorporated samples demonstrated a favorable shift, ranging from 20% to 50%, indicating a positive impact on fatty acid composition. A discernible reduction in hardness was observed with increasing oleogel substitution, notably in the 40% and 50% substitution variants. However, 10% to 30% substitution levels effectively maintained the desired texture profile of Harbin red sausages. While elevated lipid oxidation levels were evident compared to the control sample, attributed to a heightened unsaturated fat content, it is noteworthy that samples with 10%, 20%, and 30% pork back fat substitution exhibited significantly reduced malondialdehyde values compared to those with 45% and 50% substitution, indicating a favorable effect on oxidative stability.

In conclusion, Harbin red sausages reformulated with PO oleogel, substituting 10–30% of pork fat, demonstrated consumer satisfaction comparable to the original while simultaneously enhancing nutritional and health attributes. These improvements were achieved without compromising texture or sensory qualities. In summary, using an EC-based oleogel presents a promising avenue for producing healthier and more nutrient-rich Harbin red sausages [48].

### 4.3. Patties

Martins et al. (2019) conducted a study to substitute animal fat with sterol-based oleogels in pork patties [26]. The researchers utilized a 60:40 (*w*/*w*) oryzanol/sitosterol ratio mixed with linseed oil to create sterol-based oleogels. These oleogels, containing an 8% (*w*/*w*) concentration of structuring agents, were employed in all experiments. The combination of oryzanol and sitosterol oleogelators follow a self-assembly system inside the liquid phase to make oleogels. However, three sets of patties were prepared: a control set (H-Co) and two sets with partial replacement of subcutaneous pork fat with sterol-based oleogels at proportions of 25% (H-25) and 75% (H-75). The physicochemical characteristics and sensory attributes of the patties were systematically analyzed. The inclusion of oleogel, ranging from 25% to 75% of the total fat content, impacted the hardness of the samples. A discernible trend indicated a decrease in hardness with an increase in the percentage of fat replacement. The study demonstrated statistically significant variations in n6/n3 ratios due to different treatments, resulting in an elevation of the healthier cholesterol fraction in the final product when substituting pork fat with sterol-based oleogels. In the sensory evaluation, acceptance and preference tests were conducted to measure consumer appeal. The findings revealed a clear preference for the control patty samples among consumers. However, patties with less oleogel (H-25) received favorable ratings from the sensory panel in acceptance and preference tests. These results underscored the feasibility of incorporating oleogels as effective fat replacers in patty manufacturing.

Canola oil was structurally modified through the incorporation of hydroxypropyl methylcellulose (HPMC), leading to the development of oleogels, which were subsequently assessed as a viable alternative to animal fats in the production of saturated fat-reduced meat patties [49]. These HPMC oleogels exhibited behavior like an elastic gel, displaying solid fat contents independent of temperature variations. Notably, they demonstrated superior resistance to oxidative processes compared to canola oil, particularly under accelerated storage conditions. Several noteworthy improvements were observed after substituting beef tallow in the meat patty formulation with HPMC oleogels at 50% and 100% replacement levels. Firstly, the cooking loss of the patties saw a significant reduction, contributing to enhanced moisture retention and overall product quality. Additionally, the texture of the meat patties became notably softer, enhancing their palatability. Furthermore, incorporating HPMC oleogels in the meat patties positively impacted the nutritional profile. Saturated fatty acid levels in the resulting meat patties containing HPMC oleogels were substantially reduced, decreasing to only 15% compared to the beef tallow-based counterparts, which contained a much higher saturated fat content of 42%. Importantly, these findings highlight that the utilization of HPMC oleogels as a replacement for traditional animal fats not only improves the healthfulness of meat products by reducing saturated fat content but also manages to preserve their sensory attributes, ensuring a pleasurable culinary experience.

The objective of a study by Agregán et al. (2019) was to assess the efficacy of *Fucus vesiculosus* extracts in prolonging the shelf life of pork patties formulated with linseed oil oleogels and packaged under modified atmosphere conditions during refrigerated storage [50]. The oleogel utilized in this investigation comprised linseed oil and a combination of two structuring agents (γ-oryzanol and β-sitosterol). Both structuring agents were dispersed by stirring until complete solubilization, along with the linseed oil, at a temperature of 80 °C for 30 min. Subsequently, the mixture was allowed to cool at room temperature until the gel formation occurred. Pork patties with linseed oil oleogel as a fat replacer were supplemented with *Fucus vesiculosus* extract (FVE) at 250 mg, 500 mg, or 1000 mg/kg concentrations. Additionally, patties containing butylated hydroxytoluene (BHT) at a concentration of 200 mg/kg, a conventional synthetic antioxidant, were included, and patties without any antioxidant (CO) were included as the control group. The physicochemical properties and sensory attributes of the pork patty formulations incorporating seaweed-based ingredients were compared with control samples formulated with oleogels, antioxidants, or BHT. Containing FVE at 1000 mg/kg concentration into pork patties effectively safeguarding the samples against oxidation. Notably, the treatment with FVE at 500 mg/kg yielded the highest sensory scores for odor in the cooked product, indicating its potential as an optimal concentration for enhancing the overall sensory attributes of the seaweed-based pork patties.

The study conducted by Gao et al. (2021) aimed to assess the physical attributes of oleogel derived from a combination of beeswax and rapeseed oil [51]. Additionally, the research investigated the storage characteristics of beef heart patties wherein all conventional beef fat was substituted with beeswax and rapeseed oil oleogel. To create the rapeseed oil oleogels, five distinct concentrations of beeswax (2.5%, 5%, 7.5%, 10%, and 12.5% *w*/*w*) were utilized. The process involved heating the oil and wax mixture in a water bath at 90 °C and stirring it at a consistent speed of 200 r/min for 45 min. The resulting oleogels were examined for their physical properties, revealing that the 10% beeswax oleogel, when cooled at 4 °C, achieved a gel state with higher L* values than those cooled at 25 °C. Consequently, the 10% beeswax oleogel at 4 °C was chosen to replace beef fat in beef heart patties. Two patties were prepared: one with 15% beef fat (control) and the other with 15% oleogel. Subsequently, the patties were vacuum-packed and stored at 4 °C. Samples were extracted for analysis at intervals of 0, 3, 5, 7, and 14 days of storage. The incorporation of 10% beeswax rapeseed oil oleogels (cooled at 4 °C) resulted in a significant enhancement in the fatty acid composition, including a reduction in saturated fatty acids (SFA) from 54.08 to 8.34 and an increase in polyunsaturated fatty acids (PUFA) from 8.88 to 34.37 g/100 g of patties. Moreover, it led to improvements in cooking loss and the nutrient ratio of the beef heart patties. However, these oleogels were observed to decrease the oxidative stability and hardness of the patties during cold storage. In conclusion, the authors determined that adding oleogels positively influenced beef heart patties’ fatty acid profiles and nutritional indices. Nonetheless, further enhancements in the texture and oxidative stability of the patties were deemed necessary [51].

### 4.4. Pâté

The primary aim of an experiment by Martins et al. (2020) was to investigate the impact of substituting pork back fat with linseed beeswax-based oleogel on the physicochemical and nutritional characteristics of pâté [18]. The rationale behind this substitution was to identify a viable alternative for reducing saturated fat content in pâté formulations. Additionally, the study assessed the sensory attributes associated with the pâté samples. To create the beeswax-based oleogel, linseed oil was structured with 8% (*w*/*w*) of a gelator, and this process was replicated for all fat replacement experiments. Beeswax was introduced into the linseed oil under stirring at 80 °C (above the wax melting point) for a minimum of 30 min. Subsequently, the resultant gels were allowed to cool at room temperature until complete gel formation occurred, requiring a minimum of 48 h. Pâté samples were formulated with pork fat replacement levels set at 30% and 60%. The incorporation of linseed oil in the form of an oleogel contributed to an elevation in polyunsaturated fatty acids (PUFAs). Consequently, the omega-3 intake potential of pâtés with oleogel incorporation was augmented, thereby enhancing the health profile of a product traditionally not regarded as a source of health benefits. The n-6/n-3 ratio analysis revealed that substituting pork back fat with oleogel yielded a pâté with a more favorable nutritional profile than the control. Furthermore, a discernible reduction in hardness and adhesivity was observed in pâtés with oleogel incorporation. In the sensory analysis, the collated data suggested that the control and sample containing 30% oleogel received the highest appreciation, with combined scores for “liked” and “liked a lot,” reaching 7 and 6, respectively. The overall score for the 30% oleogel replaced pâté sample remained notably positive, considering its proximity to the control sample results [18].

A study by Gómez-Estaca et al., 2019, focused on structuring a more healthful lipid mixture comprising olive, linseed, and fish oils using two distinct oleogelators, namely EC and beeswax [39]. The aim was to assess the suitability of these structured lipid mixtures as functional ingredients for pork liver pâtés, with a comprehensive analysis of their physicochemical, sensory, and nutritional attributes. The researchers initiated the experiment by preparing a mixture of olive oil, linseed oil, and fish oil, combined with EC (11 g/100 g oleogel) and sorbitan monostearate (3.67 g/100 g oleogel). This mixture was then heated in Pyrex beakers within a thermostatic bath set at 170 °C under continuous stirring. After reaching a temperature of 160 °C, the mixture was allowed to stand for 10 min. Notably, the beeswax oleogel demonstrated a more rigid, ordered, brittle structure with a melting point of around 55 °C. In contrast, the EC oleogel exhibited a softer, more deformable structure with high conformational flexibility and thermal stability. Subsequently, five pâtés with a reduced fat content of 15% were formulated, with a control batch prepared using pork back fat as the primary fat source. The experimental pork liver pâtés were successfully formulated by either total or partial substitution of back fat with beeswax oleogel or EC oleogels. These reformulated products exhibited an optimal fatty acid profile, characterized by a high PUFA/SFA ratio and a low n-6/n-3 ratio, indicating their potential health benefits. Significantly, the technological behavior of the reformulated products did not significantly deviate from the control sample, including characteristics such as color and texture. Beeswax oleogel had no discernible impact on sensory parameters, while EC oleogel exhibited a negative effect directly correlated with the substitution level. Nevertheless, all samples received ratings near “neutral” on the sensory scale. Due to their composition and favorable technological and sensory properties, the developed ingredients emerge as promising candidates for producing healthier pork liver pâtés [39].

The aim of research by Barbut et al. (2021) was to explore the progressive substitution of pork fat with oleogels at varying concentrations (20%, 40%, 60%, 80%, and 100%) [52]. The primary objectives were to address the issue of oil separation and assess the impact on the sensory and textural attributes of pâtés. The oleogels were meticulously prepared in an oven at a constant temperature of 140 °C, utilizing EC with a viscosity of 20 cP, canola oil, and GMS. The gel preparation involved using glass beakers in a bench-top gravity convection oven set at 170 °C with continuous mixing at 175 rpm. The gels achieved the desired temperature in approximately 50 min, followed by a 10 min holding period. Sensory analysis revealed that complete and partial substitution of pork fat exhibited no perceptible differences compared to pâté made exclusively with 100% lard. This similarity extended to attributes such as hardness, oiliness, cohesiveness, and perceived off-flavors. The results of objective back extrusion indicated that a complete replacement of up to 100% pork fat was feasible without altering the textural characteristics of the pâtés. Optimal textural properties were maintained even with a 100% replacement of pork fat, as demonstrated by the objective back extrusion results. Furthermore, the sensory analysis suggested that a significant degree of pork fat replacement, up to 60%, could be achieved without compromising the oil retention performance while simultaneously preserving the overall textural qualities and minimizing effects on sensory attributes in the context of liver pâté [52].

### 4.5. Meat Batters

Oleogels, with their unique structural and functional properties, have demonstrated a positive impact on meat batters—mixtures of meat, fat, and other ingredients used in the production of various meat products. Oleogels contribute to the improvement of the texture in meat batters. The transformation of liquid oils into oleogels facilitates the modulation of textural attributes, providing a desirable mouthfeel that mimics traditional fat profiles. Further, oleogels contribute to the structural stability of meat batters, maintaining integrity during processing, cooking, and storage.

The aim of research by Alejandre et al. (2019) was to examine the impact of completely replacing animal fat in meat batters by employing two structured oil systems: O/W hydrogelled (HG) emulsions and oleogels [53]. Additionally, the influence of varying amounts of carrageenan in O/W hydrogelled emulsions and glycerol monostearate (GMS) in oleogels was investigated concerning the physicochemical, textural, and nutritional properties of the reformulated products. In summary, the oil phase, comprising canola oil (40%), polysorbate 80, BHT (0.01%), and kappa carrageenan (1.5% or 3%), along with deionized water (up to 100%), was individually heated to 80 °C. Simultaneously, oleogels were formulated using 12% EC, GMS (at 0%, 1.5%, or 3%), BHT, and canola oil (88%, 86.5%, or 85%), subjected to heating in an oven at 140 °C. Subsequently, seven batter samples were created, including beef fat, canola oil, two meat batters containing HG with 1.5% or 3% carrageenan, and three meat batters containing oleogels with different GMS concentrations (0%, 1.5%, and 3%). Canola oil exhibited undesirable attributes, facilitated by structuring the oil as oleogels or hydrogelled emulsions. These gel systems enhanced the meat batters’ overall quality and positively influenced the fatty acid profile. There was a notable decrease in SFA from 11.8% to approximately 2% and a corresponding increase in PUFA from 0.3% to about 5%. Furthermore, these systems demonstrated efficacy in reducing lipid oxidation. A comparative analysis revealed that oleogels were more efficiently integrated into the meat matrix than hydrogelled emulsions. Oleogels exhibited a more uniform microstructure and did not incur fat losses during the cooking process of meat batters. This research underscores the potential of structured oil systems in enhancing meat products’ nutritional quality and oxidative stability, with implications for developing healthier and more sustainable food formulations [53].

Ferrer-González et al. (2019) aimed to characterize textural differences in meat batters through compression, shear, and incision [54]. These experimental meat batters were prepared by replacing pork back fat with pumpkin seed paste and soybean oil oleogel. The investigation also included the assessment of instrumental color, changes in the fatty acid profile, lipid stability, and sensory acceptance. The soybean oil oleogel was prepared by combining EC and α-cellulose (11%, *w*/*w*) with 3.67% (*w*/*w*) Span^®^ 60 as a surfactant, dissolved in 85.33% (*w*/*w*) soybean oil. The solution underwent heating at 120 °C at a constant rate for approximately 20–30 min until complete cellulose solubilization. Simultaneously, hulled pumpkin seeds were macerated in tap water overnight to remove the green cuticle, facilitating the creation of a seed paste. The soaked seeds were cold ground to extract the oily phase. Ground seeds (63%) were mixed with water (32%), and maize starch (5%) was added as a plasticizer. The mixture was agitated until a homogeneous paste was achieved. Meat batters were prepared, incorporating 20% lard (control), oleogel, or pumpkin seed paste. Those with fat replacers exhibited a darker and less red appearance but a more yellow hue, attributed to incorporating vegetable oil. Soybean oil oleogel significantly increased the PUFA content while maintaining a total fat content close to the control sample. On the other hand, Pumpkin seed paste increased PUFA but concurrently reduced caloric content due to its lower fat content. Consumer preferences leaned towards the pumpkin seed paste samples, irrespective of the color difference and lower fat content. Significantly, the fat replacers employed to replace pork back fat substantially modified the fatty acid profile, decreased lipid oxidation, and did not adversely affect texture or overall acceptance. This suggests that these alternatives could be viable for developing healthier meat products [54].

This study, as outlined by Barbut and Marangoni (2019), aimed to investigate the implications of substituting animal fat in meat products with oleogels derived from various liquid vegetable oils [55]. The experimentation involved the utilization of beef fat and rendered beef fat in both their original and oleogel states. Oleogels were created by incorporating 10% EC with a viscosity of 10 cP, fat/oil, and 5% SMS. The gel preparation process entailed heating in an oven at 140 °C, cooling to 20 °C, covering with aluminum foil, and overnight storage at 5 °C. Meat batters were subsequently formulated to uphold 11.5% protein and 25% fat, aligning with standard retail compositions. Within the overall fat content of the meat batter formulation, 4.5% originated from lean meat. In comparison, the remaining 21.5% was derived from added beef fat, liquid canola/soy/flaxseed oil, or an EC oleogel based on vegetable oil. Transforming liquid vegetable oils into oleogels reduced batter hardness to levels akin to traditional beef fat batters. Comparing the oleogels with the native fat/oil counterparts, it was observed that the springiness of the meat batter decreased for all oleogels. Additionally, fat globule size in the oleogels produced from vegetable oils surpassed that of native oils, though this discrepancy was not evident in the case of beef fat. Adopting oleogel technology is an appealing prospect for processors and consumers. This is attributed to the capacity to manufacture products featuring elevated unsaturated fatty acid levels, aligning with the growing preference for healthier alternatives in the market [55].

The primary aim of an experiment by Shao et al. (2020) was to explore the impact of fat/oil type and EC concentration on pork batter’s texture, cooking loss, color, and microstructure [56]. The study utilized four distinct vegetable oils—sunflower seed, peanut, corn, and flaxseed—structured with EC to replace traditional pork fat in preparing pork batter. Additionally, a control group was established using pork fat to provide a benchmark for comparison. Four vegetable oils and three different concentrations of EC (8%, 10%, or 12% *w*/*w*) with a viscosity of 50 cP were incorporated into 50 g of each oil to prepare oleogels. The mixtures were then stirred at 140 °C with a rotation speed of 800 rpm until complete dissolution of EC. Post-stirring, the samples were cooled to room temperature and stored at 5 °C for subsequent use. Thirteen pork batter treatments were formulated, with pork fat as the control. Three meat batters were prepared for each vegetable oil, each with varying EC concentrations (8%, 10%, or 12%). An evaluation of emulsion stability revealed an enhancement in all oleogel groups compared to the pork fat group, with the type of vegetable oil influencing this stability. Microstructure analysis indicated smaller fat globules in batters prepared with oleogels, indicative of a stable emulsion system. Comparatively, batters formulated with pork fat exhibited larger fat globules and a lower L* value than those prepared with oleogels, though the redness (a* values) showed no significant difference. Notably, oleogels containing sunflower oil emerged as the optimal choice for pork batter in this study. Given the identified advantages, particularly in reducing saturated fatty acids inherent in traditional pork fat, oleogels are a promising substitute within the meat industry to replace animal fat [56].

**Table 1:** Application of different oleogels in meat-based products and their outcomes.

Meat Products	Unsaturated Fat	Oleogelator (%)	Gelation Conditions	Level of Saturated Fat Replacement	Effect of Oleogel Incorporation	References
**Burgers**						
Pork Burgers	Olive, Linseed, and Fish Oil	Ethylcellulose or Beeswax (11%)	160 °C or 65 °C for 10 mins	Pork fat/Partially or 100%	Significant enhancement in the PUFA/SFA ratio and a considerable drop of n-6/n-3 ratio. Beeswax oleogel-infused burgers received higher results in the sensory acceptability test.	[39]
Beef burger	Sesame oil	Beeswax (5, 7.5 or 10%)	70 °C	Beef fat/0, 25, and 50%	A substantial decrease in the hardness, gumminess, and chewiness of the raw burgers, amounting to less than 50% of the control sample. Increasing oleogel concentration in the burger correlated with a 1.5% reduction in fat absorption.	[41]
Pork Burgers	Olive, Linseed, and Fish Oil	Ethylcellulose or Beeswax (11%)	160 °C or 65 °C for 10 mins	Pork fat/100%	The oleogel enhanced the PUFA content with a significant increase in ALA, EPA, and DHA contents, respectively. Beeswax oleogel-incorporated burgers were rated more acceptable.	[42]
Beef burger	Sesame oil	Ethylcellulose (10%)	170 °C	Beef fat/0, 25, and 50%	The EC oleogel mitigated the oxidation process during frozen storage and reduced cooking loss and fat absorption in beef burgers. Additionally, the introduction of EC oleogel has led to enhancements in textural properties, specifically in terms of chewiness and hardness.	[43]
**Sausages and Frankfurters**						
Breakfast sausage	Canola oil	Ethyl cellulose (8, 10, 12 or 14%), and Sorbitan monostearate (1.5 or 3.0%)	140 °C for 50 min	Pork fat/20.8%	Exhibited lower objective hardness and springiness ratings but comparable cohesiveness to controls containing pork fat and canola oil. However, incorporating SMS into the oleogels resulted in objective hardness values closely resembling those of the pork fat control treatment.	[38]
Frankfurter Sausages	Soybean oil	Rice Bran Wax (2.5% or 10%)	90 °C for at least 30 min.	Pork back fat/17.67%	Enhanced fatty acid profile and textural attributes like firmness, and chewiness in both 2.5 and 10% RBW incorporated samples. A 10% RBW addition had unfavorable effects on the flavor and overall acceptability.	[44]
Frankfurter Sausages	Linseed Oil	Beeswax (8%)	80 °C for at least 30 min.	Pork back fat/25 or 50%	There was a significant reduction in SFA content, leading to a favorable impact on the n-6/n-3 ratio and a decrease in cholesterol content. However, it is noteworthy that specific sensory parameters, including color, did not exhibit substantial improvement.	[45]
Bologna type sausages	Sunflower oil	Pork Skin (37.50%)	80 °C for 40 mins	Pork back fat/25, 50, 75, and 100%	Substitution levels of up to 50% demonstrated superior nutritional and technical attributes while maintaining sensory characteristics and without concurrent increases in lipid oxidation comparable to the control sample.	[46]
Semi-Smoked Sausages	Sunflower Oil	Monoglycerides and beeswax (20%)	90–95 °C for 60 min with stirring at 150 rpm.	Pork fat/7 or 10%	The content of PUFA and MUFA increased, improving the overall fatty acid profile while maintaining good sensory properties.	[47]
Harbin Red Sausages	Peanut Oil	Ethylcellulose (6%, 8%, 10%, or 12%)	140 °C with stirring at 800 rpm	Pork back fat/10,20,30,40, or 50%	Demonstrated consumer acceptance on par with the original formulation at 10–30% substitution. This modification improved nutritional profile and health characteristics without compromising the sausage’s texture properties and sensory attributes.	[48]
**Meat patties**						
Pork patty	Linseed oil	Oryzanol and *β*-sitosterol (60:40) (8%)	80 °C for 30 min	Pork fat/25% or 75%	Resulting in an elevation of the healthier cholesterol fraction in the final product when substituting pork fat with sterol-based oleogels. In addition, patties with a lower amount of oleogel (25%) received favorable ratings from the sensory panel in both acceptance and preference tests.	[26]
Beef patty	Canola oil	Hydroxypropyl methylcellulose (2, 4, or 6%)	400 rpm for 3 mins	Beef tallow/50 or 100%	Saturated fatty acid levels in the resulting meat patties containing HPMC oleogels were substantially reduced, decreasing to only 15% compared to the beef tallow-based counterparts, which contained a much higher saturated fat content of 42%.	[49]
Pork patty	Linseed oil	*ɣ*-oryzanol and *β*-sitosterol (60:40)	80 °C for 30 min	Pork fat/5%	Incorporating oleogels into pork patties proved effective in safeguarding the samples against oxidation. Notably, the treatment yielded the highest sensory scores for odor in the cooked product.	[50]
Beef heart patty	Rapeseed oil	Beeswax (2.5%, 5%, 7.5%, 10%, or 12.5%)	90 °C for 45 °C with stirring at 200 rpm	Beef fat/15%	Improvement in cooking loss and the nutrient ratio of the beef heart patties. However, these oleogels were observed to decrease the oxidative stability and hardness of the patties during cold storage.	[51]
** Meat Pâté **						
Pork pâté	Linseed oil	Beeswax (8%)	80 °C for 30 min	Pork fat/30% or 60%	The n-6/n-3 ratio analysis revealed that substituting pork back fat with oleogel yielded a pâté with a more favorable nutritional profile than the control. The sample containing 30% oleogel received the highest appreciation, with combined scores for “liked” and “liked a lot” reaching 7 and 6, respectively.	[18]
Pork liver pâtés	Mixture of olive, linseed, and fish oils (44.39%–37.87%–17.74%)	Ethyl cellulose (11%), Sorbitan monostearate (3.67%), or Beeswax (11%)	160 or 65 °C at speed 3	Pork back fat/9% or 15%	Exhibited an optimal fatty acid profile, characterized by a high PUFA/SFA ratio and a low n-6/n-3 ratio, indicating their potential health benefits. Significantly, the technological behavior of the reformulated products did not deviate substantially from the control sample, including characteristics such as color and texture.	[39]
Pork pâté	Canola oil	Ethyl cellulose (12%) or Sorbitan monostearate (3%)	140 °C for 50 min with stirring at 175 rpm	Pork fat/20%, 40%, 60%, 80%, or 100%	Sensory analysis revealed that both complete and partial substitution of pork fat exhibited no perceptible differences compared to pâté made exclusively with 100% lard. This similarity extended to attributes such as hardness, oiliness, cohesiveness, and perceived off-flavors.	[52]
**Meat Batter**						
Beef batter	Canola oil	Ethylcellulose (12%) and glycerol monostearate (0%, 1.5%, or 3%)	140 °C and kept in the oven at 100 °C for 1 h	Beef fat/22.84%, 23.23%, or 23.63%	There was a notable decrease in SFA from 11.8% to approximately 2% and a corresponding increase in PUFA from 0.3% to about 5%. Furthermore, these systems demonstrated efficacy in reducing lipid oxidation.	[53]
Meat batter	Soybean oil	Ethyl celluloses mixture (11%) and Span^®^ 60 (3.67%)	120 °C for 20–30 min	Lard/20%	Soybean oil oleogel significantly increased the PUFA content while maintaining a total fat content close to the control sample. Significantly, the fat replacers employed to replace pork back fat substantially modified the fatty acid profile, decreased lipid oxidation, and did not adversely affect texture or overall acceptance.	[54]
Beef batter	Canola, Soy, and flaxseed oil	Ethyl cellulose (10%) and Sorbitan monostearate (5%)	140 °C for 50 mins with stirring at 200 rpm	Beef fat/21.5%	The transformation of liquid vegetable oils into oleogels facilitated the reduction in batter hardness to levels akin to traditional beef fat batters. Additionally, fat globule size in the oleogels produced from vegetable oils surpassed that of native oils, though this discrepancy was not evident in the case of beef fat.	[55]
Pork batter	Sunflower, Peanut, Corn, or Flaxseed Oil	Ethylcellulose (8%, 10%, or 12%)	Stirred at 140 °C with 800 rpm	Pork fat/100%	Improved emulsion stability of all oleogel-infused samples. Sunflower oil oleogel had enhanced textural parameters, including color, and exhibited better emulsion stability.	[56]

## 5. Advantages of Using Oleogels in Meat Products

Oleogels present a compelling alternative to animal fats in meat products, offering advantages beyond simple fat substitution. Notably, their incorporation of liquid vegetable oils elevates the nutritional profile of meat items, reducing the reliance on traditional animal fats. This addresses concerns related to saturated fats in diets abundant in unsaturated fatty acids, promoting a healthier fat composition in the final products and aligning with consumer preferences for healthier food options.

The shift towards incorporating oleogels in meat product formulations is substantiated by numerous experimental findings. These findings, conducted by researchers in the field, offer a foundational reference for the meat industry to adopt this alternative formulation, driven by both nutritional and technological considerations. Recognizing that hard fat is a natural reservoir of trans-fats, with processed meat products reportedly containing an average of 35% saturated fats, underscores the significance of this shift in light of the prevalent health concerns associated with increased cardiovascular diseases among consumers [57,58].

A study by Gómez-Estaca et al. (2019) exemplifies the positive impact of oleogels in meat burger development, utilizing EC and beeswax oleogels with a healthy lipid mixture as fat replacers [39]. The reformulated burgers displayed a significantly improved fatty acid profile, indicating a 3.6-fold increase in the PUFA/SFA ratio and a 23-fold decrease in the n-6/n-3 ratio compared to the control. Similarly, applying soybean oil oleogels structured with rice bran wax in Frankfurter-type sausages resulted in enhanced fatty acid profiles, characterized by higher essential polyunsaturated fatty acids and lower saturated fatty acids than the pork fat control [44]. Furthermore, Martins et al. (2020) utilized linseed oil oleogels structured with beeswax to enhance the nutritional profile of pâtés, demonstrating an increase in polyunsaturated fatty acids and a substantial decrease in the n-6/n-3 ratio. Oleogels contribute significantly to improving the texture of meat products by transforming liquid vegetable oils into modulated textural attributes, providing a desirable mouthfeel akin to traditional fat profiles [18]. A previous study by Zetzl et al. (2012) underscores the impact of oleogels on the overall mouthfeel of meat products, enhancing the eating experience through desirable textures such as smoothness, creaminess, or juiciness [59].

Oleogels also contribute to the structural stability of meat products, as demonstrated by Barbut et al. (2016), who found that HPMC oleogels exhibited enhanced textural properties and reduced cooking loss in meat patties, enhancing their overall quality [38]. This structural stability extends the shelf life of meat products by mitigating textural and sensory deterioration and lipid oxidation. Panagiotopoulou et al. (2016) experiments revealed no statistically significant differences in lipid oxidation levels between control and oleogel-formulated Frankfurter treatments after 30 days of storage [60]. Oleogels act as a physical barrier, shielding the oil from oxygen exposure and reducing the likelihood of oxidation, with some gelling agents possessing antioxidant properties. Oleogels enable processors to tailor properties based on specific meat product requirements by offering formulation versatility, as evidenced by studies conducted by Oh et al. (2019) and Martins et al. (2020) [18,49]. These studies indicate that the overall acceptability of meat products can be maintained or even improved by replacing traditional animal fats with oleogels derived from liquid vegetable oils. Oleogels transcend their application in heat-resistant foods, presenting versatile solutions to issues such as oil leaks and serving as carriers for lipid-soluble bioactive chemicals [2].

In conclusion, incorporating oleogels in meat products yields multifaceted benefits, encompassing improved texture, an enhanced nutritional profile, structural stability, and increased consumer appeal.

## 6. Challenges in Replacing Saturated Fats in Meat Products

Replacing saturated fats in meat products presents multifaceted challenges, primarily focused on retaining desired sensory qualities while meeting consumer expectations and nutritional considerations. The distinctive flavor and aroma of meat products derived from saturated fats pose a significant hurdle in finding suitable replacements replicating these characteristics.

Sensory analysis has highlighted significant preference differences (*p* < 0.05) influenced by substituting pork back fat with linseed oleogel. Control samples consistently garnered higher favorability scores for odor and taste, emphasizing the challenge of replicating the sensory experience with oleogel substitutions. Despite a slightly higher hardness observed in the 25% substitution sausage, control samples received superior global perception scores compared to 25% and 50% oleogel substitutions [45].

Saturated fats also play a pivotal role in the texture and mouthfeel of meat products, impacting tenderness, juiciness, and overall texture. Achieving a comparable or improved texture proves complex, as seen with EC oleogels causing decreases in color, taste, and overall acceptability values in pâtés. These changes varied with the oleogel quantity present, indicating the intricacies of balancing sensory attributes during fat substitution [39].

Manipulating instrumental textural hardness values with SMS-containing oleogels shows promise, as previous data indicates, but aligning them with sensory perception requires further exploration. Producing harder oleogels, as suggested by Gravelle et al. (2014), may enhance sensory hardness sensation [61]. Although juiciness values of oleogel treatments were lower than pork fat Frankfurters and canola oil control due to better oil retention within the oleogel structure, challenges persist in improving other sensory parameters such as color and cohesiveness [38]. These challenges demand additional research for enhanced sensory properties and lipid oxidation in precooked products utilizing linseed oleogels [45]. Notably, samples with substitutions exceeding the 50% level experienced a marked decline in overall acceptance, underscoring the delicate balance between innovation and sensory expectations in modified Bologna sausages [46].

Furthermore, saturated fats exhibit more excellent stability against oxidation than unsaturated fats. Substituting them with unsaturated or modified fats may increase susceptibility to rancidity and off-flavors, as evidenced by a significant increase in lipid oxidation, particularly with EC oleogel at the highest substitution level during refrigerated storage [39]. Developing strategies to enhance the oxidative stability of meat products with reduced saturated fats is imperative. Balancing nutritional value while reducing saturated fats and potentially increasing unsaturated fats or other beneficial components poses a challenge. Altering fat composition can impact processing parameters, such as emulsification and stability, introducing challenges on an industrial scale. Ensuring that reformulated products maintain quality and sensory attributes over time is crucial.

In response to these challenges, ongoing research and development efforts are concentrated on identifying and optimizing suitable fat replacements, refining processing techniques, adjusting oleogel substitution percentages, and addressing consumer education and perception regarding healthier meat products.

## 7. Conclusions and Future Research

In conclusion, recent policy adjustments mandating the elimination of trans fats from food items and restrictions on the intake of saturated fats, coupled with growing consumer apprehensions regarding the adverse effects of fats and the environmental repercussions of extensive palm oil usage, have spurred a surge in studies exploring the substitution of pork back fat and beef fat. Traditionally, natural biopolymers, cereal flours, dietary fibers, and proteins have been investigated for their potential to reduce fat content and enhance meat products’ oil and water retention capabilities. However, these alternatives fail to replicate animal-derived fats’ flavor, sensory experience, and nutritional aspects.

A promising solution appears to be oleogel, serving as a solid fat substitute due to its capacity to impart textural and mechanical properties akin to conventional fats in food products while simultaneously enhancing nutritional profiles. Oleogel production involves straightforward processes utilizing the oleogelation technique, employing both direct and indirect methods to immobilize the liquid phase within a three-dimensional network and transform it into a solid, gel-like structure with the aid of oleogelators. Moreover, the structuring agents employed necessitate minimal concentrations, accessibility, and affordability.

Structured lipids are introduced into meat products as alternatives to animal fat, requiring careful consideration of their composition and physicochemical characteristics, as these factors influence the quality of reformulated products. A comprehensive understanding of structured lipid characteristics is crucial, facilitating their incorporation, elucidating their role in the protein matrix structure, and enhancing the quality of the healthy, meat-based food systems they improve.

Given the novelty of oleogel technology in the food industry, extensive research is imperative to evaluate its positive effects and potential drawbacks, such as gelators’ acute and chronic toxicity effects. A nuanced comprehension of oleogels’ characteristics, their ability to control phase separation, and their impact on decreasing the mobility of the oil phase is vital for developing innovative applications. Investigating various oleogel formulations considering component types, ratios, and properties suitable for meat products is an ongoing necessity. Additionally, the issue of compatibility in food products should not be overlooked, thus ensuring the commercial viability of oleogel-based food products as a healthier choice for consumers in the future.

## Data Availability

The data presented in this study are openly available in the article.
